# Vulgar things: Moral dilemmas of luxury consumption in an unequal society

**DOI:** 10.1177/14695405251376100

**Published:** 2025-08-31

**Authors:** Brandaan Huigen

**Affiliations:** 1 Department of Anthropology, 4919 University College London, London, UK

**Keywords:** conspicuous consumption, redistribution, morality, luxury, social inequality

## Abstract

There are discussions in South Africa, the world’s most unequal society, about the morality of consuming and displaying luxuries. Anger is especially directed at political elites eager to display their expensive trappings, while others can barely survive. This article considers the negative consequences of conspicuous consumption in South Africa by analysing instances of discontent being directed at political elites and their possessions of luxury brands. I argue that such luxuries can become ‘vulgar’ in unequal South Africa especially when consumed by political elites thought to be hypocritical, when positioned within socioeconomic crisis and when regarded as the proceeds of theft. Ultimately, luxuries that remain detached from effective redistribution, which helps to soften tensions that arise from social hierarchies in human societies, become egregious. Finally, I consider why, despite their vulgarity in South Africa, many political elites continue to publicly display luxury goods.

## Introduction

There are often public debates in South Africa about the consumption habits of political elites. Their expensive tastes and possessions, often products of global brands, like *BMW* vehicles, *SMEG* appliances, *Michael Kors* clothing and *Cîroc* vodka, are morally questioned: What gives these influential people the right to consume such luxuries? How dare they rub such luxuries in the faces of poor South Africans? Do they have no shame?

Ever since democratisation in the 1990s, after centuries of white-minority rule, African National Congress (ANC) politicians and their associates have amassed substantial wealth, not only through their high-salary jobs, but also because of corporate interests and lucrative government contracts that are often irregularly awarded to friends and family (Cotterill, 2017). To the displeasure of many, these political elites seem to have no qualms with flaunting their trappings,^1^ that a large proportion in the world’s most unequal society could only dream of affording. There are, however, also counter voices – not least arising from these owners of wealth themselves – who say that there is nothing questionable about consuming luxuries in an unequal society. In fact, displaying such objects, and aspiring to own them, should be worthy of respect (Huigen, 2025).

These contestations surrounding certain objects and their display by political elites in South Africa, is the focus of this article, a topic which concerns broader discussions on the morality of conspicuous consumption, specifically in an unequal socioeconomic setting. The aim is to analyse how and why certain luxury goods come to be regarded as ‘vulgar things’, as morally questionable valuables when possessed by certain people in South Africa. While anthropological and sociological scholarship on consumption culture in Africa, in particular, is interested in analysing materialistic lifestyles (see Van Wyk and Deborah, 2019), there has been a general reluctance to consider the morality of acquiring and displaying contemporary luxury goods. Being critical of flashiness is found to be “patronising”, doing little to challenge “Eurocentric assumptions” in consumption theories about what luxury is and how it comes to attain value in African contexts (Dosekun and Mehita, 2020: 46). Another view holds that criticism of consumption, more generally, regards consumer cultures around the world as becoming increasingly homogenous, Westernised and trite. These totalising critiques from the late 19^th^ and 20^th^ centuries (see Adorno and Horkheimer, 1972; Veblen, 1994; Baudrillard, 2016) are thought to limit the agency that local populations have in creatively integrating popular commodities into their lives, including elite members of society, which will have ambiguous and unique sociocultural effects depending on the setting (Miller, 2001). Contrasting with this view is a totalising perspective which takes as universal given that conspicuous consumption, specifically, is a progressive step in the natural development of nations, especially when transitioning from low- to high-income status (Postrel, 2008). Deprived populations that have embraced mass consumerism after long periods of communist, colonial or kleptocratic rule, tend to find conspicuous consumption important as a way of offsetting the low social status that poverty or previous oppression brought (see Friehe and Mechtel, 2014). The idea is that, almost inevitably, populations will eventually embrace “inconspicuous consumption” as they become richer, moving to the consumption of ‘quiet’ over ‘loud’ luxuries. Especially political elites, in countries like China, feel pressure to consume quiet luxuries (Osburg, 2013).

It is certainly necessary not to dismiss these standpoints, and to belittle the love for luxury goods as superficial. In fact, luxury consumption in its diverse forms builds close bonds and alliances, and is a sign of experimentation with new elite cultures in transitioning societies, as I have also argued before (Huigen, 2017). But it is equally important to take seriously the public outcries about conspicuous consumption and when this is thought to become egregious; that there are moral contradictions and destabilising *social* consequences to consider with the consumption of luxury goods in certain contexts, especially when consumed by people with access to state resources. This is a point that analysts have overlooked, opting to view conspicuous consumption as perhaps lacking tact and provoking irritation, but which would fundamentally remain a socially benign practice. Socialist theorists and politicians, such as Thomas Sankara in Barkino Faso, or Kwame Nkrumah in Ghana, have however questioned materialistic lifestyles. They viewed an excess of luxury, especially when held in elite circles, as having negative consequences and that the ideal of a more equal society, with fewer material disparities, is worth striving for (see Nkrumah, 1970).

This article takes an anthropological perspective on the morality of luxury goods consumption and display without necessarily aligning to socialist ideologies, though understanding such concerns about inequality. It suggests that in unequal societies such as South Africa, luxury consumption and display become vulgar without adequate redistribution – the sharing of collective wealth – which should be performed by political elites. This has serious negative consequences that can harm a population, not only on a symbolic level, but by keeping large segments impoverished and increasing crime and violence.

After a brief discussion on why certain objects are required in human societies to signal and shape status, causing wealth disparities which need to be regulated through redistribution systems, I will look into the violent consequences of luxury consumption in South Africa. This is followed by the presentation of social media outcries concerning political elites – specifically about the Minister of Electricity, Kgosientsho Ramokgopa wearing a *Michael Kors* sweater, and the then deputy president of the Economic Freedom Fighters (EFF) while wearing a *Karl Marx* shirt – displaying these objects to the public. The analysis section that follows reflects on the cases through three interrelated themes, showing why certain luxuries become ‘vulgar’ in the South African context, and morally repugnant. This seems especially to occur when luxuries are consumed by hypocritical political elites, when positioned in a society suffering from socioeconomic crises and when regarded as the proceeds of theft. I conclude with a discussion on the need in anthropology, and consumer studies more broadly, to take seriously the negative consequences of certain consumption practices when these remain uncoupled from effective redistribution. I further reflect on possible reasons why, despite the vulgarity of luxury in South Africa there is nevertheless little effort among political elites to embrace inconspicuous consumption.

For the above argument, I draw on 16 months of ethnographic fieldwork in Cape Town, South Africa, between 2017 and 2018, and another period in 2024, which focussed on property theft and illicit forms of consumption. However, considering events in the social media space, specifically on X, theoretical insights from fieldwork are complemented by a discourse analysis of X posts, which revealed relevant themes for the discussion. This social media platform offers the possibility to observe how status objects are displayed to the public, while revealing how powerful people and the public react to each other (Ligaga, 2012).

## Objects and hierarchies in human societies

Throughout the modern human past, certain objects have served to reflect and produce social hierarchies, an inevitable structural characteristic of *homo sapiens* societies. While loosely organised at first, social status ranking, including leadership positions and political ranking, has become formalised and permanent in small- to large-scale societies (Plourde, 2009). Objects of display have served the same purpose all over the world for the last five millennia: to signal and enhance social status (Clark, 1986). These are typically objects of adornment made from (relatively) rare materials that can be worn, carried or placed in relation to certain people, such as heirloom jewellery and statues. Kwakiutl coppers, Māori cloaks and axes, the golden Rhinocerous of Mapungubwe and *kula* armshells and necklaces are examples of this, which were considered the most valuable objects in the respective cultures (Graeber, 1996). In many African cultures, however, precious status objects are not only produced from inanimate materials – such as gold, pearls, wood or ivory – but also include living animals like cattle. As Comaroff and Comaroff (2005) note, cattle had the capacity to enhance the wealth of owners, becoming a supreme form of property in sub-Saharan Africa. The milk, meat and blood from the cows ensured the biological reproduction of the group, while their exchange for bridewealth built social relations and political alliances within and between groups.

This is a feature of status objects more generally: they do not only ‘statically’ signal certain badges of office, skills, titles and leadership roles, as earlier anthropologists such as Radcliffe-Brown thought (Tilley, 2006), but they actively produce wealth through exchange opportunities, inheritance and the shaping of alliances. They are in the league of ‘inalienability’ (Curasi et al., 2004), which elite kinship groups (i.e., chieftainship, nobility or royalty) or particular social groups (i.e., the leaders of political parties), who often control the broader society through formal institutions, come to possess. Whether crown jewels or inherited mansions, these aesthetically unique objects reflect and produce social hierarchies through gift exchange. Reflecting the identities and biographies of their successive owner(s), these objects stand in contrast to mass-produced commodities of contemporary capitalist societies, which Marx (1976) thought of as anonymous material bundles of alienated labour, that are bought and sold on the market.

Anthropologists have, however, questioned a neat distinction between Marxian alienable commodities and Maussian inalienable gifts (Herrmann, 1997), and the social relations that imbricate their respective materialities. Global consumer products of certain brands can also become inalienable, depending on how they are circulated and absorbed inside and between groups. An expensive luxury vehicle or watch bought on the market, can signal status and be inherited as a family heirloom (see Huigen, 2025; Kessous et al., 2017; Kopytoff, 1986). But the point remains that certain objects come to represent and produce hierarchies in human societies, having distinctive material features depending on the technological and historical epoch they are produced, while others carry less social and aesthetic weight.

## Luxury, inequality and redistribution

Especially sumptuous material cultures, which luxuries are typically thought to fall under (Dosekun and Mehita, 2020), display the distinction of certain classes over others. There are, however, debates about whether luxury items are always defined by conspicuous qualities, with recent research suggesting an increasing decoupling in many societies of luxury from conspicuousness and even real wealth (Eckhardt et al., 2015). Luxury may be associated with more sophisticated tastes and subtle aesthetic cues that only a select group of people would understand. Notwithstanding, luxury has, at least until recently, been associated with noticeable materialities (Silvia et al., 2018) and graphics, which purposefully bring attention to the possessor and their power. This remains especially important for a growing *nouveau riche* class in developing countries like South Africa, who want to signal their rise into wealth to compensate for a previous lack of it (see Rucker and Galinsky, 2008). Unfortunately, the conspicuous display of luxuries to signal status contrasts sharply with the coarse and nondescript materialities of poverty in South Africa.

To soften and legitimate such social-material divides that luxury products produce, every society requires systems of redistribution. In small-scale societies these instruments were the potlatch feasts of the Pacific north-west indigenous groups, for example, with taxation, welfare and various development schemes in large-scale societies having a similar purpose: to pool and fairly distribute resources in and between groups and regulate social imbalances. The institutions of chieftainship and modern nation states are the central nodes that are commonly responsible for redistribution in society (Firth, 2013; Sahlins, 1974). While efficient redistribution implemented by chiefs or public representitives does not completely eradicate the distinction of more powerful groups, it does seem to make visible wealth more bearable for the broader population. Elites in France, Japan and increasingly Rwanda, for instance, may well find ways to demarcate their social status, through art and other ‘refined’ or ‘quiet’ forms of inconspicuous consumption for instance (see Bourdieu, 1984; Eckhardt et al., 2015), but their display of certain luxuries will hardly have broader social implications in such generally egalitarian societies, where high-ranking possessions are coupled with effective redistribution. Through the principles of sharing and reciprocity on a societal scale, luxury items, and the social status that they represent are buttressed from negative consequences. Crucially then, redistribution is at the heart of morally legitimating the consumption and display of luxuries in society.

This is not the case, as the next section will show, in societies like South Africa where redistributive systems hardly function or have completely collapsed, leading to uncontained forms of consumption and luxury possession that become vulgar and offensive to the general public. Such anger is not without justification, for when luxuries attain vulgar status, they can cause harm in society.

## Consequences of conspicuous consumption

South Africa is the most unequal country in the world (SSA, 2019). Though regarded as a middle-income country on official statistics (Seekings and Nattrass, 2008), more than half of South Africans live in poverty, most of whom are black and brown people. Since the end of apartheid, the lower-middle- to upper classes have become increasingly diverse and integrated, coming close to Nelson Mandela’s hope of creating a ‘rainbow nation’. The further hope was, in Mandela’s spirit, that South Africa would become a country grounded in the African principle of *ubuntu*, in which caring and sharing are core values that citizens and the state should uphold (Metz, 2011).

Yet the fractured nature of South Africa has persisted along socioeconomic lines, owing, on the one hand, to a history of colonialism and apartheid, which redistributed wealth for a white minority at the expense of the black majority, and on the other hand, owing to largely failed redistribution schemes of the ANC-led government since democratisation in the 1990s. Especially since the Jacob Zuma Presidency between 2009 and 2018, the latter mechanism virtually formed a kleptocracy, in which political elites siphoned vast resources from the public purse for themselves and their patronage networks (Cotterill, 2017). These resources, which were meant to uplift the lives of poorer South Africans, have instead sustained the conspicuous lifestyles of a politically-connected minority (Huigen, 2017).

Stealing money from the public purse, sourced from South Africa’s limited tax base, for the consumption desires of the political elite has negative consequences, the most obvious being that redistribution does not take place. Money that is meant for quality infrastructure, healthcare, electricity, housing, education, policing, social security and job-creation does not effectively reach poorer South Africans. They remain impoverished and vulnerable without wealth being channelled to them. This leads to increased inequality and resentment towards those – not only the political elite – who possess visible wealth.

Apart from feelings of resentment and envy (see Belk, 2011), an even more dangerous and overlooked phenomenon stems from this situation. Marginalised South Africans, especially, create their own underground and violent systems which enforce redistribution. This is most commonly achieved through two types of acquisitive criminality: property theft and extortion.

Property theft in South Africa, specifically robberies or burglaries, is mostly committed by marginalised men targeting individuals who possess expensive branded luxuries, whether iPhones, flatscreen televisions, expensive cars or fashionable clothing. Once stolen, sometimes after killing the owner, in the majority of cases these items are channelled into impoverished neighbourhoods, often to low-income residents who the thieves know (see Huigen, 2024). These luxuries are subsequently redistributed through the underworld, with thieves often justifying their crimes with Robin Hood myths (see Hobsbawm, 1972). Similarly, extortion is a form of criminality which has become increasingly intrenched as South Africa became more unequal. With extortion, well-resourced individuals or organisations, often state organisations, are pressurised by extortionists through violent means to share lucrative contracts or profits. The proceeds are often spent on luxury consumer goods and lavish lifestyles.

Luxuries therefore become ‘costly signals’ (Cannon and Rucker, 2019) and contested in a highly stratified society without centralised redistribution, causing envy among lower-income populations (see Belk, 2008) who are not benefitting from redistribution, further attracting violent crimes that enforce decentralised forms of ‘levelling’ disparities caused by, amongst other things, the display of luxuries. These crimes are underground ways to consume luxuries that are typically out of financial reach for indigent people. As luxury goods are frequently targeted by violent criminals, owning conspicuous luxuries can be extremely dangerous in South Africa. While a poor thief might justify stealing these items and murdering the owner by claiming that past and present injustices have marginalised him and has not received the benefits of employment or educational opportunities – as his similarly marginalised receivers of the stolen goods – it is difficult to see how political elites can justify stealing collective resources meant for upliftment. This is especially considering that they are the ones responsible for giving marginalised segments of society the means to generate wealth by redistributing tax money. Simply put, political elites often commit reverse Robin Hood: they steal from the poor to give to a rich, politically-connected, clique. Even if political elites did not steal public funds, their lavish lifestyles would remain questionable as they are connected to a state that has failed to redistribute wealth.^2^

These dilemmas of luxuries not linking well to effective redistribution open up questions amongst the South African public of the morality of especially political elites possessing luxuries in an unequal society, as the next section presenting two cases of a government minister and a radical politician will show. With these cases, we see that there are important arguments raised in the public sphere for and against the conspicuous display of luxuries amongst South Africa’s political elites, which need to be understood in relation to the tense historical and present situation facing the country. These arguments will be analysed in the section that follows to determine why luxuries become vulgar.

## Questionable luxuries

### ‘Minister of Michael Kors’

When the energy crisis deepened in South Africa in March 2023, leaving South Africans with no electricity for large parts of the day, President Cyril Ramaphosa appointed Dr Kgosientsho ‘Sputla’ Ramokgopa, an ANC politician, as the new Minister of Electricity. With a PhD in Public Affairs, and as a former mayor of South Africa’s capital city, Tshwane – where he was involved in a tender scandal and illicit mansion makeover (Brederode, 2023) – the hope was that he would quickly gain control over the failing coal stations and criminal syndicates controlling Eskom, the national power utility. During the first 3 months of his tenure, Minister Ramokgopa seemed energetic about solving the crisis. Yet little changed the situation, with power cuts becoming even worse. Indeed, the public increasingly came to regard Minister Ramokgopa as someone with big promises but no real action. He already had a reputation as someone who loved partying, but did not seem serious about government work (see Nkanjeni, 2003). However, his appearance on the news channel ENCA, where he was interviewed once again about his plans, caused outrage.

**Figure 1.**
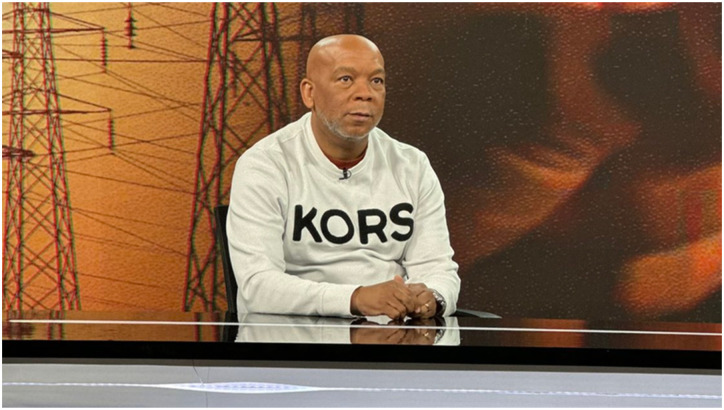
Minister Ramokgopa displaying a KORS sweater while talking about the energy crisis on television. Source: X/Kgosientsho_R

The outrage on social media was caused by the Minister posting an image of himself on X wearing a casual sweater with large bold black letters reading KORS (see Figure 1) – the exclusive label of the American fashion designer Michael Kors (see Ramokgopa, 2023). X-users were quick to point out that this particular KORS sweater costed over R6000 (about 350 US dollars). The majority of commentary directed at the Minister questioned the wearing of this KORS sweater, while a minority defended the Minister by engaging his detractors. There were three main themes of comments. First, many detractors questioned why he could not wear more suitable clothing for his role and the event. For example:

A1: Let me guess he couldn’t wear a shirt and jacket because of loadshedding [blackouts]. Helper was not able to iron shirts.

B1: He must wear some thing [sic] plain. In an economy like ours this Michael Kors sweater is a bit Marie Antoinette.

C1: Dress shirt and jacket (maybe a tie) would be better than being a Michael Kors billboard.

D1: Read the room. I’m not saying don’t wear your fav brands. But your PR team has failed here. You are here to address an issue that’s crippling the economy daily, and the attention is on the indirect endorsement of a luxury brand .... Out of touch.

E1: Grootman [Big man]. Next time please wear local brand. That sweater looks so wrong.

D1: We know and understand you can afford to wear any clothing brand but appearing like this on a National tv is an insult to the poor.

F1: A plain shirt or golf shirt would do. I know this was on the weekend but he really could have worn something else. This looks tacky like you’re not reading the room

G1: He should have wore [sic] a suit or something not branded. That tshirt [sic] was distractive nje. He doesn't take his audience serious.

The defenders of the Minister noted, often through hyperbolic and absurd alternatives, that the display of the sweater was not morally unsound. For example:

A2: Ministers must have uniforms? [includes meme of confused face]

B2: But he’s a minister, what must he wear? Something from small street [informal traders selling cheap counterfeit clothing]?

C2: I really don't under the furor [sic] with his sweater, he works, and can afford things he likes, so he must shop at Pep to make you bitter souls happy? … Let the man spend his money how he sees fit!

F2: A minister earns over R2million a year... All his clothing is expensive. When he comes there in a suit, we know its NOT from Markhams.

G2: Ahhyi yall are reaching... You want young leaders, when you have them you complain that they don't dress like Manthashe... Does it even matter?? Should we not be focused on what he is saying and how he does his job. A sweater???

Second, the detractors often questioned the means by which the conspicuous sweater, and his wealth and that of other ANC ministers in general, were acquired. For example:

A3: Did not expect anything less from one of South Africa’s thieving ministers.

B3: That's PEU meters money...TshweneEntso ke lehodu [transl. from SeSotho: he is a thief]!

C3: Thieves wear designer clothes because taxpayers foot the bill.

A typical response to these accusations, especially when levelled by a white person, was as follows:

A4: His [sic] a minister he can afford it. Buy him clothes dat u [sic] deem appropriate…for him… N [sic] tell your oupas [white Afrikaner grandfather] n [sic] oumas [white Afrikaner grandmother] to bring back the stolen land.

Third, there were many snide remarks and threats of civil unrest that were telling of the disgust that [mostly black] South Africans feel towards ANC politicians who display luxuries. For example:

A5: Next year we say goodbye to ANC [meme of a crying woman]

B5: We hate you.

C5: Eat, comrade! Eat!

D5: Wena [Hey you] you must resign; Voetsek [go away]!

E5: Streets are going to burn

F5: Let them eat cake.

### ‘Real revolutionaries wear Karl Marx’

The second case study concerns Floyd Shivambu when he was a member of the Economic Freedom Fighters (EFF), a radical left, populist party. He is known for his expensive tastes, similar to the ‘Commander-and-Chief’ of the EFF, Julius Malema. Shivambu was part of the EFF for more than a decade before moving to the MK party, a period in which he was seen, alongside Malema, as embracing the consumption of expensive Western brands. This was despite forming part of a party guided by Marxist, anti-West and Pan-Africanist revolutionary ideologies, claiming solidarity with the poor by wearing miner’s overalls and gumboots during parliamentary sessions. In passionate speeches, the EFF leadership promote to radically redistribute the wealth owned by white and Indian South Africans, the country’s more fortunate ethnic groups. However, while being Members of Parliament since 2013, Shivambu, Malema and their families have tremendously benefitted from other financial sources. There were serious allegations of them having defrauded the VBS mutual bank (see Van Wyk, 2019). Shivambu, for his part, has allegedly bought luxury vehicles, expensive clothes and properties with the proceeds of this alleged crime.

**Figure 2.**
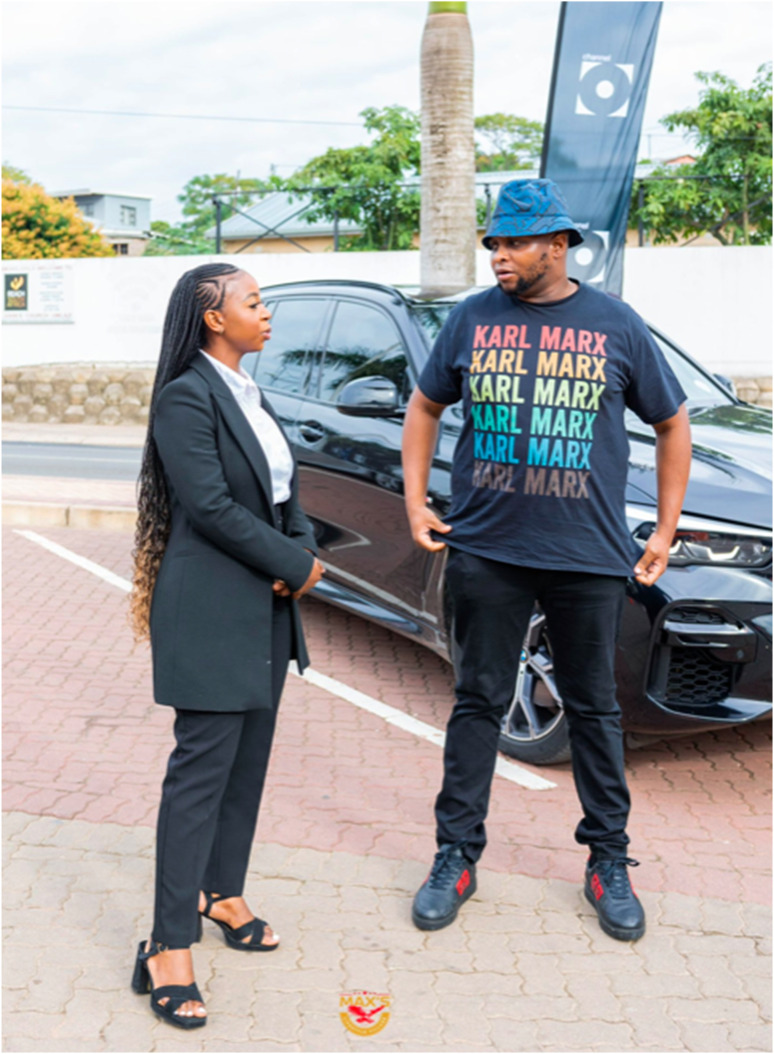
Floyd Shivambu (right) donning a Karl Marx shirt and Gucci sneakers in front of the BMW X5 at Max’s Lifestyle Village. Source: X/maxslifestyle1

On June 2023, a day before the ‘Minister of Michael Kors’ fracas on social media, Max’s Lifestyle Village, a bespoke ‘kasi’ (township) restaurant and hospitality business in Durban – where political elites were often seen partying – posted a photo of a famous visitor “gracing us with his presence”. Shivambu can be seen chatting to the establishment’s manager in front of a BMW X5, which detractors claim to be his. Apart from wearing expensive Gucci sneakers and pants, he was wearing what seems to be a designer shirt with a recurring motif in rainbow colours of the name ‘Karl Marx’ (see Figure 2), who EFF members idealise. The irony of the merchandise was not lost for many. As one detractor remarked (Max’s Lifestyle Village, 2023): “Karl Marx that drive around on [sic] R1.5 million BMW and R72k sneakers.” There were three main themes of comments, starting with this topic that Shivambu’s trappings were tainted by a seemingly hypocritical lifestyle (as was the case with the Minister). For example:

A6: Look at me standing in front of my car. Is a nice car. Look at me sporting all the imperialists' swag. I only talk, but I do not mean a word I say.

B6: CEO [lifestyle], but a supporter of Communism. How even?

C6: Why don’t he drive China cars since they don’t love Europe? … He must refuse it and let GWM [Chinese brand] come and escort me.

D6: Question is where are his stinky gumboots and dirty Red overalls? This clown and his role model [Malema] are opportunists. Imagine claiming to represent poor masses yet driving around a R3 million Rand SUV wearing a R70k sneaker. Honorable CLOWN this one.

Counter-points noted that Shivambu could spend his hard-earned money how he wished, while others were simply upset with those questioning Shivambu’s hypocrisy:

A7: If [his] salary allows the affordability then no problem with that because he has a right to use his salary the way he likes. It’s his sweat.

B7: It’s sad that black witches and jealous white scotrums [sic] have camped here to spew bile.

The second theme was where detractors made more explicit reference to the contradictions of the Karl Marx shirt:

A8: We don’t love whites but we love Karl Marx [who was a white man].

B8: The name of Karl Marx V/S [versus] the price of the sneakers he is wearing.

C8: Did Karl Marx teach materialism? Looks like DP Shivambu is a materialistic Marxist who subscribe to consumerism. Critical of capitalism but enjoying the fruits of capitalism.

D8: Real revolutionaries wear Karl Marx.

Hyperbolic examples as counter-points were similarly made as with the Minister’s defenders, such as the absurd proposal of Shivambu being expected to wear a cheap “PEP” shirt instead. The final theme related to Shivambu’s alleged crimes and defrauding of VBS mutual bank:

A9: Just pay back the VBS loot you stole… So-called communists that love the drapings [sic] of the capitalists.

B9: Forgot to mention that he is a VBS looter.

C9: Skinny jean givin sum baggy vibes….VBS money eatin him up.

D9: Spending the VBS money.

E9: Fat Floyd and Brian [Shivambu’s brother] are thieves.

Counter points mainly professed Shivambu’s innocence – that if he had really stolen millions from the ‘Gogos’ (grandmas), he would have been prosecuted by now.

## Vulgar things

In South African society, questioning conspicuous consumption is particularly common owing to the country’s contentious history and persisting and even worsening inequalities; in a context of both scarcity and plenty. Especially in this context, global luxury products become morally questionable. But their vulgarity is further amplified, as the above exemplary cases illustrate, when political elites supported by state salaries ostentatiously show them off, causing public outrage. This section will reflect on why this is the case, arguing that luxury goods become vulgar when they are displayed by hypocritical political elites, when flaunted during times of crisis and/or considered as the proceeds of theft. In closing, we need to answer an important question: if luxuries become morally illegitimate and vulgar owing to the aforementioned reasons, especially by remaining uncoupled from effective redistribution, why do political elites persist in flaunting these luxuries?

### Blingy officials

The thrust of much criticism directed at Minister Ramokgopa and Shivambu was that they were public representatives while displaying conspicuous luxuries. As left-leaning politicians who were supposed to represent the interests of poorer South Africans, their extravagant lifestyles were thought to not be in line with their ideological/discursive commitments, which are underpinned by the principle of (radical) redistribution, even if, as in the case of Shivambu, they do not hold office in government, but are members of parliament or councillors. In both cases they are still funded by the public purse to oversee that a more equitable society will materialise. In this regard, luxuries become vulgar because they express ideological contradictions and the hypocrisy of the owner: a disjuncture exists between what they say as political figures with redistributive roles, and what is materially signalled.

In the case of Minister Ramokgopa, the KORS sweater exemplified this disjuncture most blatantly, to the extent that he was regarded as an advertising “billboard” for an American fashion label (e.g. C1), rather than being committed to his state responsibilities. Comments therefore indicated that it would have been more appropriate for the Minister to wear less flashy, locally-produced or plainer clothes commensurate with his high status as a Minister and the public event of appearing on television (e.g. A1, B1, F1). Those who defended the Minister, provided extreme and unrealistic examples of dress expectations to make his critics seem silly, saying that the Minister was expected to wear cheap and fake-branded clothing bought from informal traders or stores catering for the working-class (e.g. B2, C2). They ignored that critics were not asking for low-status dress, but a reasonable dress standard that symbolised responsibility and professionalism (e.g. a “suit and tie”), which the loud bold letters of KORS, as a conspicuous brand, and the general casualness of a sweater, did not display. This further indicated for many a growing conflation of the state with the fashion/leisure industry, with politicians wanting to be noticed as celebrity icons, rather than focussing on being diligent state workers. This view is reminiscent of Veblen’s observations on the extravagance and excesses of the upper ‘leisure class’, who would rather spend their time on status competitions to show-off their wealth through luxuries, than work hard (Veblen, 1994).

Similarly, with Shivambu the contradiction between political ideology and the symbolism of certain objects was clear with his wearing of the multi-coloured Karl Marx shirt, which was regarded as a fashion item. Similar to what Stallybrass (1998) notes about Marx’ coat, the shirt exemplified the contradictions of commodity fetishism in capitalist societies. In this regard, the shirt was viewed in relation to Shivambu’s extremely expensive BMW and Gucci sneakers. These items were not commensurate with his, and the rest of the EFF leadership’s political commitments; of standing in solidarity with the poor and to realise an equitable society (e.g. B6, D6), but it instead demonstrated their appetite for luxury commodities. Fetishizing commodities, the excessive desire to acquire objects that are the products of alienated labour, is at the heart of Marxist critique in unequal capitalist societies (see Marx, 1976), like South Africa. This is part of a wider irony: politicians claiming to be Marxist revolutionaries and anti-Western, yet obsessed with consuming Western luxury brands — the very things they are supposed to be fighting against. Instead, Shivambu is seen displaying items that symbolise and perpetuate extreme social rifts between the elite and low-income citizens. Indeed, EFF defenders ridicule the Sankarist idea of ‘dressing down’ to be in solidarity with the poor (see Peterson, 2021), which may be expected of socialist politicians and elites more generally (see Osburg, 2013). However, the EFF only engages in this behaviour for political reasons, especially during parliamentary sessions or election campaign marches. This is not the case in their everyday lives, which is the real domain of constructing cultural identities (see Baudrillard, 2016), where luxuries are consumed for status competitions between political elites; at ‘lifestyle villages’ for instance.

### ‘Whilst Rome burns’

The contradictions of displaying luxuries as public representatives, is not merely questioned on a symbolic level, but represent serious failures of governance, especially the failure of elite individuals buttressing their luxuries with effective redistribution. This failure leads to social malaise. As noted earlier, luxury consumption and display may be legitimated in society if the collective deed of redistribution, of sharing wealth, is effectively executed. A failure at achieving this goal does not only lead to animosity towards political elites for keeping and exacerbating the impoverished circumstances of those who rely on them, but also to general animosity towards private individuals who own substantial wealth (cf. Belk, 2011). When political elites are sanctioned as wealth redistributors, they also do so on behalf of the middle- and upper-classes through their tax contributions. Even if these classes want to have their wealth redistributed, their luxuries also become vulnerable and vulgar owing to state inefficiencies.

But specifically, the Minister’s failures at redistribution, and that of political elites more generally, were correlated with the ongoing electricity crisis that South Africa has been facing, where the national power utility, Eskom, almost collapsed in 2023 owing to ANC-affiliated officials and associates being unable to manage the company, and partly due to the diversion of public funds (see next section). The lack of electricity supply has hit poorer South Africans the worst, because they are solely reliant on the state for services; wealthier South Africans can afford to buy solar panels. The purported incompetency of the Minister at his job, forms part of growing frustrations with the ANC government’s inefficiency during the post-apartheid era, failing to deliver quality education, infrastructure, healthcare, security, housing, often contracting family, friends and crooked business men instead of experts. This has led to anger amongst those who rely on basic services, with frequent protests breaking out, often directed at the property of representatives (i.e., their luxury homes and cars).

This anger was also directed against the Minister when displaying his KORS sweater, which reminded many of the tangible failures of governance as a result of incompetence and corruption. Many critics consequently threatened to start violent protests (E5) and never to vote for the ANC again (A5), or simply expressed their disgust, adding that the Minister should resign for wearing the KORS sweater (B5, D5). The KORS sweater was considered inappropriate because South Africa was in a state of crisis – “whilst Rome burns” – as a direct result of failing redistribution. Money spent on the sweater could have been used for upliftment. The sweater was considered a hedonistic and excessive display of wealth, which was not collectively legitimated through the reallocation of wealth, having real consequences for South Africans either remaining or entering a life of poverty and despair, worsening social inequality. In this regard, the individual in relation to the object became vulgar for failing to “read the room” (e.g. D1); for being callously ignorant of the malaise and those who suffer as a direct result of the ANC’s redistribution failures.

### Eating and stealing

A final subject of criticism for displaying luxuries, was that these objects are often alleged or proven to be consumed through the proceeds of theft, and therefore situated in the moral economy of stealing funds meant for redistribution. Both the Minister and Shivambu are alleged to have financially benefited, respectively, from a tender and looting scandal. These funds were used to support their luxury lifestyles that could hardly be supported through a relatively modest state salary, even as a high-ranking Minister or MP. Their critics consequently joked that the comrades “eat” too much (e.g., C9, C5, F5).

This is a common phrase used on platforms in South Africa to indicate how socialist politicians and their business associates plunder public resources in a gluttonous manner, which is not only a jab at how overweight many become after benefitting from multi-million-rand plunders that make them obscenely rich, but their blatant disregard to share public resources meant for upliftment. This is a phenomenon that is not unique to South Africa. In many postcolonial African societies political elites divert public funds for private gain, with the public making snide remarks about gastral excess to emphasise especially their material obscenity (Bayart, 2009). In this way, political elites come to embody the power of the state within the “imaginary of the belly and eating, the right to capture and the redistribution of spoils – all these being metaphors common in local vernaculars of power” (Mbembe, 1992:8).^3^

Recall that redistribution is fundamentally about sharing pooled wealth to maintain stability in society, making social inequalities less extreme and tolerable. The cannibalisation of public resources by those who are meant to execute this sharing, and their often-unashamed capture and distribution of these spoils within a closed clique of political elites, rather than the rest of society, is the converse of building constructive hierarchies in society. Instead of building a more equitable society through the act of giving (Mauss, 2002), the transfer of theft takes without giving back (see Narotzky and Moreno, 2002; Newell, 2006). This is not a reciprocal deed, but damages relations between deprived and elite segments of society. Political corruption, the use of powers by government officials and their networks to divert public funds, is therefore considered a criminal and shameful act in most societies that requires (severe) retribution (Hunt, 2005). Yet political elites in South Africa have managed to ‘legitimate’ plunder, further eroding the criminal justice system to prevent their imprisonment.

## Concluding discussion

On the basis of two exemplary cases, this article focussed on the moral dilemmas of consuming and displaying luxury items in the world’s most unequal society. Luxury items, whether intricate art pieces, clothes, shoes, watches or vehicles of outstanding brands, are supposed to represent and produce the important roles, responsibilities and abilities of their possessors, structuring hierarchies between people. Perhaps no role is more important for a society than that of the political leaders responsible for redistributing pooled wealth and achieving this complex task of creating a more equitable society. But when these sanctioned persons have failed at this task, by simply being ineffective, and/or stealing pooled wealth for themselves and associates, their trappings become the cause of discontent. When one group feels materially deprived compared to another, discontent can lead in democracies to a change of government through voting, but also to criminal forms of redistribution. In extreme cases, unchecked disparities can lead to revolutions and even massacres (see Mamdani, 2001).

In South Africa, when material wealth is not adequately shared, this leads to highly skewed property relations, and those who possess luxury items, are, in the very least, questioned. I have consequently argued that these discussions about the morality of owning luxuries in an unequal society, that are raised in the public sphere, need to be taken seriously, and not dismissed as trivial frustrations or mere ‘jealousies’. The consumption and display of certain valuables can become vulgar for the general populace, especially when many remain uncoupled from to effective redistribution, which should bring the required agency to build wealth, thereby softening disparities. Luxury goods that are not buffered by centralised sharing, make them glaring, contrasting strongly with the effects and materialities of failed governance: the coarseness, dysfunctionality and desperation of grinding poverty. In this context, luxuries like KORS sweaters, BMW ‘beemers’ and Gucci sneakers are not things of ‘humility’ (see Miller, 2005) or ‘inconspicuousness’ (Eckhardt et al., 2015). With their consumption and overt visibility in unequal South Africa, they rather become things of vulgarity with violent effects, that are embedded in fractious historical and social relations.

But why, considering their acute impact on social harmony, do especially political elites continue to shamelessly display vulgar things? Why do they feel that they have this right? This is especially considering findings that wealthier classes in certain developing and developed countries may prefer to practice inconspicuous consumption precisely because they do not want to provoke envy and anger in times of hardship (Belk, 2011; Bourdieu, 1984).

There are three main reasons why I believe political elites in democratic South Africa stubbornly persist with public displays. First, most are part of the *nouveau riche* class, often after having formed part of the struggle against apartheid. This combination supposedly sanctions conspicuous consumption, for not only are ANC/EFF/MK politicians and their social networks often the first generation to access substantial wealth through politics and the state – a generation that is more generally susceptible to compensatory consumption to make-up for perceived material deficits (Rucker and Galinsky, 2008) – but they were part of the anticolonial resistance movement. This resistance was, as Robert Ross (2023) correctly observes, not only a fight for equal human rights, but also to reach the trappings of privileged South Africans with generational wealth, which oppressed groups were deprived of with colonialism and apartheid, justifying the present ‘it’s our turn to eat’ discourse and practice at this relatively early stage of democracy following authoritarian rule (cf. Friehe and Mechtel, 2014). Despite the immorality of stealing public resources, present injustice therefore often extends to questions of historical injustice in South Africa, as in other postcolonies. The logic is that if past ‘eating’ occurred during colonialism and apartheid, which were systems that I helped to sacrificially dismantle, present ‘eating’ may occur to spoil myself for causing revolution. Secondly, when having the means to display luxuries, political elites become obsessed with narrow status competitions between themselves in which these objects play a crucial role in building sociality and distinction, trumping concerns of the wider social impact of luxuries beyond their own cliques and geographies of glamour (see Huigen, 2017), where yells of anger and envious stares are easily lost at lifestyle villages and walled-off mansions. Finally, as I have shown, while there are detractors, there are also many who idolise the rapid rise of individuals and their hedonistic displays. These celebrants are seduced by luxury ownership, associating excess with dignity and comfort. As has been observed elsewhere (see Pinheiro-Machado, 2010), this perception is common amongst low-income citizens, who seek to emulate the trends of wealthier classes, to the extent that necessities may be sacrificed to acquire status objects (sometimes even through the stolen goods market). Owning such luxuries allows a form of escapism from otherwise difficult circumstances and offsets feelings of relative deprivation. They are, ironically, also the people who keep figures like Shivambu and Ramokgopa in power, who will, predictably, feel exalted by their jubilations during the rare moments when they make contact with the masses during election campaigns, for instance. Their normalisation of luxury display, unfortunately, sets the extremely high bar for these aspirant classes, who mimic these trends, even with little financial resources.

## Notes

1 In this article I focus on ‘political elites’, a term that refers in South Africa not only to individuals who hold formal authority within society, like politicians, judges and councillors, but a blurred network of people who benefit from their proximity to state resources (see Bayart, 2009), including business people, family members of politicians and socialites.

2 Furthermore, the lavish lifestyles and luxuries of upper-class white South Africans is also considered questionable, as they benefited from effective — albeit discriminatory — redistribution in the past. This is an important topic of discussion, but will not be covered at length here considering the scope of this article.

3 Beyond Africa, there are, of course, many other societies where (dictatorial) kleptocracies reign, like Russia, Venezuela and Myanmar. But bodily metaphors relating to the theft of public resources seem to be quite unique to the African context.
